# Association of strength and plyometric exercises with change of direction performances

**DOI:** 10.1371/journal.pone.0238580

**Published:** 2020-09-10

**Authors:** Hallvard Nygaard Falch, Håvard Guldteig Rædergård, Roland van den Tillaar

**Affiliations:** Department of Sport Science and Physical Education, Nord University, Levanger, Norway; Federation University Australia, AUSTRALIA

## Abstract

The change of direction (COD) ability is an important task-specific skill for success in team sports, dependent on both strength and reactive strength. The sprint approaching the COD and degrees of the turn are factors influencing the specificity of the COD. Thus, CODs have been suggested to be categorized as force- (> 90°) and velocity-dominant (< 90°) dependent on the degree of the turn. When training programmes fail to provide a significant increase in COD performance, it is often due to neglecting the task-specific demands of the COD. As such, 23 male football players volunteered to complete a randomized controlled trial, investigating the association of maximal strength and power performance with performance in a force- (180°) and velocity-dominant (45°) COD, with a 4 m and 20 m sprint approach. Three strength and three plyometric exercises, matched in movement patterns, were used. Muscle activity of the different conditions was also compared. The correlational analysis revealed that better performance in the plyometric tests were associated with less time to complete both force- and velocity-dominant CODs, supported by similarities in muscle activation. None of the performances in strength exercises correlated to COD performance, due to the slow contraction velocity of maximal lifts. It was concluded that plyometrics share more physical similarities with CODs than the strength exercises.

## Introduction

Field sports require a set of different physical abilities to succeed in competition [1–3], such as the ability to move fast with change of direction to overcome the opponent, which largely influences overall performance [4]. These fast movements are important due to the short duration of match-decisive moments, and may be the difference between scoring or conceding a goal [5–8]. Without accounting for perception, the term used in research terminology is the change of direction (COD) ability [9]. The COD is dependent on the athletes’ physical ability to accelerate, decelerate and re-accelerate into a new direction [10, 11]. COD ability has been found in earlier research to be a distinguishing factor between elite and sub-elite athletes [7, 12, 13]. Since improvements in COD ability may lead to an overall greater performance in field sports [14], how to improve the decisive physical components of COD performance is dependent on is of interest to strength and conditioning coaches. CODs are fast dynamic movements, where the objective is to exert as much force as possible, in a short duration of time. Thus, maximal strength, reactive strength and power of the lower limbs are factors thought to influence COD performance [15].

As such, strength- and plyometric-training interventions are often utilized to try to improve COD performance, implementing different squat variations performed bilaterally and unilaterally, with movements in both the vertical and lateral direction [16–22]. Plyometric training often includes drop jumps [23–27] and/or countermovement jumps [28–33] with variations in ground reaction forces produced. The effect of the different training interventions varies, but failing to induce large and significant effects is often due to a lack of specificity in movement patterns between the chosen exercises and the test selected for measuring COD performance [34]. Often neglected is the task specific nature of COD ability [35], dependent on number of turns, angle of direction change and sprint distance covered [10, 34, 36, 37]. Therefore, exercises utilized to improve COD performance must be similar in movement patterns, direction of force production and time to exert force, as argued in a review by Falch, Rædergård [34]. Bourgeois, McGuigan [36] also argued for considering the distinctiveness of different CODs, emphasizing velocity approaching the turn, and degrees of the turn. Furthermore, CODs were suggested to be categorized as force- (>90°) or velocity-dominant (<90°) based on the degree of the turn [36].

Strength training is suggested to be most effective for developing force-dominant CODs, while plyometric training is suggested to be more effective in developing the spectrum of qualities influencing COD performance [34]. However, to the best of the authors’ knowledge, no study has specifically investigated the similarities of performance measures in strength and plyometric exercises with the proposed force- and velocity-dominant CODs. Therefore, the objective of the current study was to investigate the relationship of performance in force- (180°) and velocity (45°) -dominant CODs from different approaching distances (4m vs 20m), with performance in different strength and plyometric tests. To better understand the relationship of exercise and COD performance, a second objective of comparing muscle activation between the different conditions was added.

The question of interest was whether performance and muscle activation in the strength tests could better account for performance and muscle activation in the force-dominant COD, and vice versa, the plyometric tests and the velocity-dominant COD. Greater knowledge about similarities in muscle activation and association in performance measures between the strength and plyometric tests when compared to force- and velocity-dominant CODs may lead to greater insight in developing more specific training programmes. Performance and muscle activation in the plyometric tests were hypothesized to better predict COD performance in both CODs, due to similarities in time to exert force.

## Materials and methods

### Participants

Twenty-three male experienced football players (age: 22.5±2.6 years, height: 181.3±6.3 cm, body mass: 79.9±8.6 kg) recruited from the second to sixth division of the Norwegian national league and participated in football training for a minimum of two times a week, participated in the study. All the participants were right-foot dominant, defined as preferred foot for kicking the ball. Each subject was informed to avoid heavy training and consuming alcohol with a minimum of 24 hours prior to testing and further procedures and risks of participation were explained to each participant. Before the tests, a signed written informed consent was provided by the participants, which was approved by the Norwegian Centre for Research Data project nr: 42440 and performed according to the declaration of Helsinki and of the journal [38].

### Experimental design

A within-subject design was employed, where all participants performed a series of different COD tests, strength tests and plyometric tests in one test occasion. This was conducted to investigate the association of performance measures in the strength and plyometric tests with performance in force- and velocity-dominant CODs. Muscle activation data were collected from the tests to investigate the similarities in activity levels between the different exercises.

### Procedures

Prior to testing, two days of familiarization was required, practising the strength, plyometric and COD tests, to avoid results at the day of testing being affected from a learning effect. A minimum of four days was set between each day of familiarization, to promote recovery and to establish one repetition maximum (1RM) in the different strength exercises. Participants were encouraged to provide maximal effort in all exercises on the familiarization days to avoid unnecessary fatiguing attempts to establish 1RM and to control whether it was their maximal effort at the day of testing. The protocol was identical for the days of familiarization and testing, starting with the COD test before the strength and plyometric tests were performed in a randomized order. First, on the day of testing, height and body mass were measured. Afterwards, electromyography (EMG) was placed at ten muscles of the dominant lower limb before dressing up in a full body motion capture suit. After all the equipment was attached, the warm-up protocol for the COD test was initiated.

The warm-up consisted of five minutes jogging at a self-selected pace, before performing three runs with increasing intensity up to 60%, 70% and 80% of self-perceived maximum intensity. Afterwards, participants performed a specific warm-up of running 15 metres, then turning either right or left with a 65° or 110° at 80% of self-perceived maximum intensity. In total, four runs (right or left: 2 x 2: 65° or 110°) were performed in a randomized order with 60 s rest between each run. After the warm-up, the COD test was performed at maximal intensity with a three-to-five-minute rest between each attempt.

**Fig 1:**
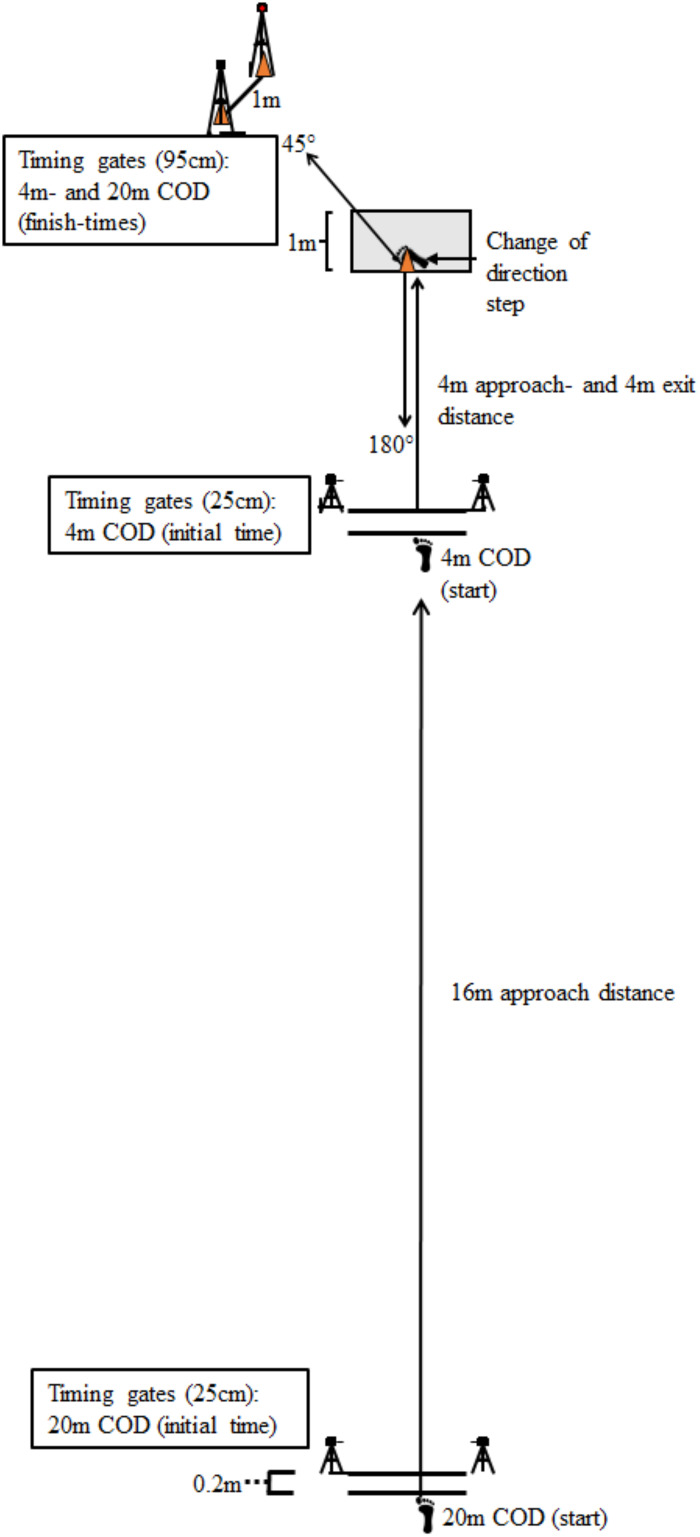
Change of direction test set up with approach of 4 or 20 m with timing gates on 4 and 20 m with COD of 45 and 180° followed by 4 m sprint.

The COD test consisted of a 45° and 180° turn with a 4 m and 20 m sprint approaching the COD, making four runs in total (Fig 1). The COD track was designed to investigate the extremities of force- vs velocity-dominant turns, while accounting for sprint distance approaching the turn. All CODs consisted of left turns, making the dominant foot (right) perform the COD step. The COD test was performed on an indoor court surface (Taraflex Sport Evolution M 7.0 mm, Unisport, Finland). Participants began from a standing-still start, with the front foot placed 20 cm behind the starting line placed either 4 m or 20 m from the COD area (Fig 1). The participant started running on their own accord with maximal effort. The time started when crossing the first timing gates placed along a two-metre-long starting line running towards the COD area, where the subject performed a left turn (45° or 180°) and re-accelerated 4 m to finish off the test. For an attempt to be approved for the 45° COD, the subject had to turn from right to left around a cone placed in the midpoint of the COD area. The cone was removed when performing the 180° COD, then both feet were required to be inside of the COD area (Fig 1) for an attempt to be approved. In case of slipping or violations of the requirements due to any other unpredicted events, a reattempt was required. Also, to ensure maximum effort, a test attempt resulting in a performance decrease of 0.1 s or more from the second familiarization day resulted in a reattempt.

**Fig 2:**
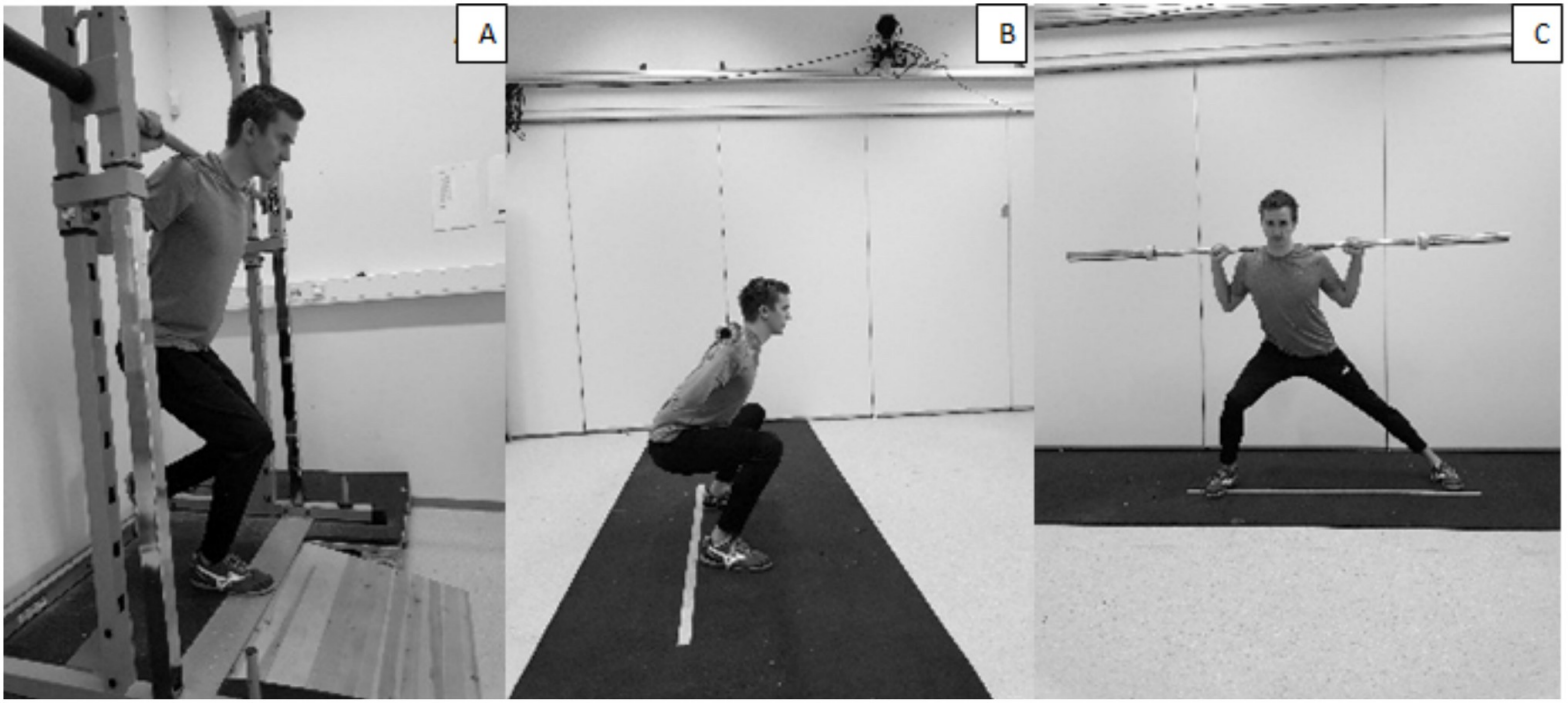
Illustration of the strength exercises. (A) Unilateral quarter squat. (B) Bilateral parallel squat. (C) Lateral squat.

**Fig 3:**
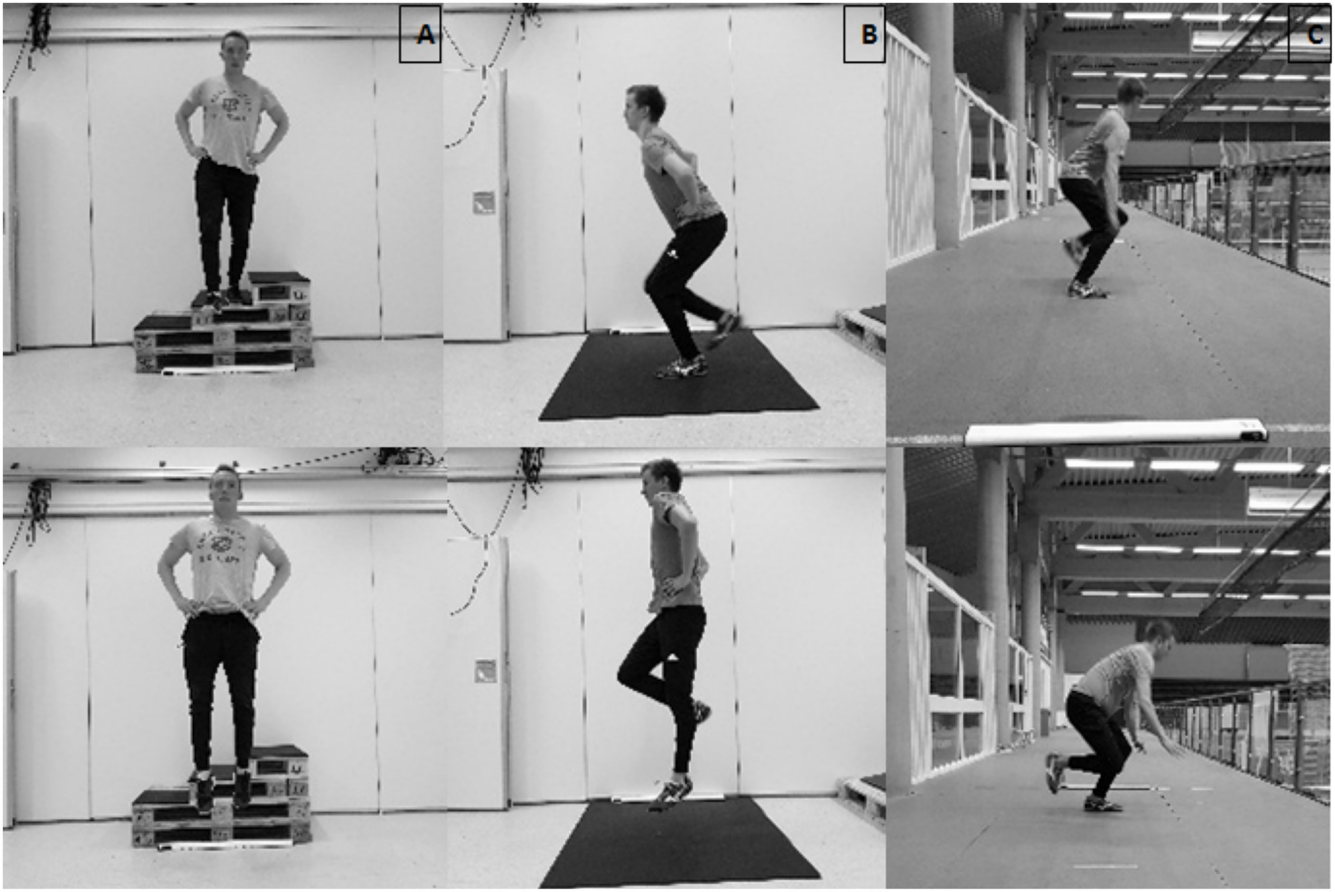
Illustration of the different plyometric exercises. (A) Drop jump. (B) Countermovement jump. (C) Skate jump.

After the COD test, the strength and plyometric exercises were performed in a randomized order (Figs 2 and 3). As the participant was already warm, the only warm-up prior to the strength and plyometric exercises consisted of performing the exercise at submaximal intensities, leading up to a maximal effort attempt. For each of the strength and plyometric tests, the participant had three attempts. However, one attempt for establishing 1RM in the strength exercises was mostly enough, due to the estimates from the second day of familiarization. Proper performance technique (mainly depth) in the different exercises was controlled for by using 3D modelling. An extra attempt was necessary if requirements of the tests were violated. The test-attempt with highest performance, within the given test requirements, was used for further analysis.

The chosen exercises conducted in the vertical direction performed bilaterally and unilaterally are often used in training interventions [10, 34] and the strength and plyometric tests were matched in movement direction. Additionally, the skate jump and lateral squat were chosen since they could simulate the lateral direction of the COD step [39]. The *parallel squat* was performed bilaterally. The depth required was that a visualized line between the patella and trochanter major should be parallel to the ground. The *unilateral squat* was performed with the dominant foot in a Smith machine and bending the knee until reaching 40 degrees. The *lateral squat* was performed by moving the dominant foot to the side, with an extension of the hip initiating the movement. The dominant foot’s heel should not be placed any further forward than the toes of the non-dominant foot. The depth of the dominant foot needed to be 90° while the non-dominant foot was required to be stretched out.

In the *unilateral vertical countermovement jump*, the performance variable was jump height. The test was performed with the dominant foot, with hands placed akimbo and a straight back, limiting usage of the back and arms to contribute to jumping performance. The non-dominant foot was required to remain passive throughout the jump. The *drop jump* was performed from an individually customized drop height ranging from 0.15 to 0.6 metres, found at the days of familiarization. Optimal drop height was the height from which the subject obtained the highest reactive strength index (RSI) value, which was the performance variable of the test (flight-time/contact time). The RSI value was obtained by a contact grid. When performing the drop jump, hands were akimbo. *Skate jump* was performed laterally, jumping with the dominant foot for maximal distance. For an attempt to be approved, the subject needed to stand still in the landing. Distance was measured manually with measuring tape to the closest 0.01 m.

### Measurements

Total time to complete the COD test was measured with electronic timing gates (Brower Timing Systems, Salt Lake City, Utah, USA). RSI and jump height were found using infrared optical contact grid (Ergotest innovation, Porsgrunn, Norway) with a resolution of < 0.02 s. The contact grid consists of two units, irSOURCE and irMirror, which reflects an infrared carpet a few millimetres above the floor. Contact time was recorded when disrupting the infrared carpet. Jump height was calculated by flight time, using the equation:

$$\text{jump}\;\text{height}=½*9.81*{\left(\text{flight}\;\text{time}/2\right)}^{2}.$$

Muscle activity was measured using EMG (Ergostest Innovation, Porsgrunn Norway) with a sampling rate of 1 kHz. The EMG sensors were attached to electrodes (Zynex Neurodiagnostics, CO, USA) on ten different muscles of the subjects’ dominant foot. The skin had to be shaved and washed with alcohol before placing the electrodes (11 mm contact diameter and 2 cm centre-to-centre distance), along the direction of the presumed muscle fibres on the lateral and medial vastii, rectus femoris, adductor longus, biceps femoris, semitendinosus, soleus, gastrocnemius, gluteus medius and maximus muscles according to the recommendations of Hermens, Freriks [40].

The EMG raw signal was amplified and filtered using a preamplifier located as close as possible to the pickup point to minimize noise induced from external sources through the signal cables. The preamplifier had a common-mode rejection ratio of 106 dB and the input impedance between each electrode pair was > 10^12^ Ω. The EMG raw signal was then bandpass-filtered (fourth-order Butterworth filter) with cut-off frequencies of 20 Hz and 500 Hz. The resulting EMG signals were converted to root mean square (RMS) signals using a hardware circuit network (frequency response 450 kHz, averaging constant 12 ms, total error ± 0.5%). Peak EMG signal in the COD step and acceleration step where the dominant foot was in contact with the ground was used for further analysis, together with the peak EMG in the strength and plyometric exercises. EMG was collected in Musclelab 10.5.69 (Ergotest innovation A.S, Porsgrunn, Norway) and synchronized with a contact grid and a 3D motion-capture system: Xsens motion capture, MVN link (Xsens Technologies B.V. Enschede, Netherlands). Xsens is a full-body motion-capture system, based on 17 inertial sensors on different anatomical points with a sample rate of 240 Hz.

### Statistical analyses

Correlational analysis was performed by Pearson’s *r*. Level of significance was set at *p* < 0.05. Strength performance for correlation was expressed by Wilks points, a valid way of normalizing strength performances by accounting for body mass [41]. Jump height/length and the reactive strength index were performance measures used for the plyometric exercises. A one-way ANOVA with repeated measures was used to compare muscle activation in the different strength and plyometric tests with the COD step, and first subsequent step of the dominant foot (acceleration step). When significant differences occurred, the Holm−Bonferroni post hoc test was conducted. Violations of the assumption of sphericity was adjusted for by the Greenhouse−Geisser correction.

## Results

The different strength and plyometric performances together with the COD times were displayed in Table 1. None of the strength tests correlated significantly with COD performance (*r* < -0.41, *p* > 0.08). Drop jump performance was only significantly correlated with the 20 m 45° COD (*r* = -0.52, *p* = 0.01), while the countermovement jump significantly correlated with both the 4 m 180° (*r* = -0.61, *p* = < 0.01) and the 20 m 45° COD (*r* = -0.49, *p* = 0.02). The skate jump performance was found to be significantly correlated with performance in all CODs (*r* > -0.49, *p* < 0.02) (Table 2).

**Table 1:** Main (± SD) performance in the different strength, plyometric exercises and CODs.

Exercise	Performance
**Strength tests**	
Bilateral squat (Wilks points)	87.3±10.3
Unilateral squat (Wilks points)	72.7±9.1
Lateral squat (Wilks points)	71.5±9.7
**Plyometric tests**	
Drop jump (RSI)	1.4±0.3
Unilateral countermovement jump (m)	0.184±0.032
Skate jump (m)	2.01±0.19
**Change of direction**	
4m 45° (s)	1.68±0.15
4m 180° (s)	2.43±0.12
20m 45° (s)	4.01±0.20
20m 180° (s)	4.88±0.24

RSI = reactive strength index.

**Table 2:** Correlation between the different strength and plyometric exercises with the different change of directions.

	4m 45°	4m 180°	20m 45°	20m 180°
**Bilateral squat**	.083	.012	-.008	-.117
**Unilateral squat**	-.186	-.138	-.097	-.165
**Lateral squat**	-.410	-.238	-.276	-.233
**Drop jump**	-.235	-.344	-.542*	-.356
**Unilateral CMJ**	-.402	-.608*	-.492*	-.080
**Skate jump**	-.491*	-.564*	-.767*	-.602*

CMJ = countermovement jump

*Indicates a significant correlation at the *p*< 0.05 level.

**Fig 4:**
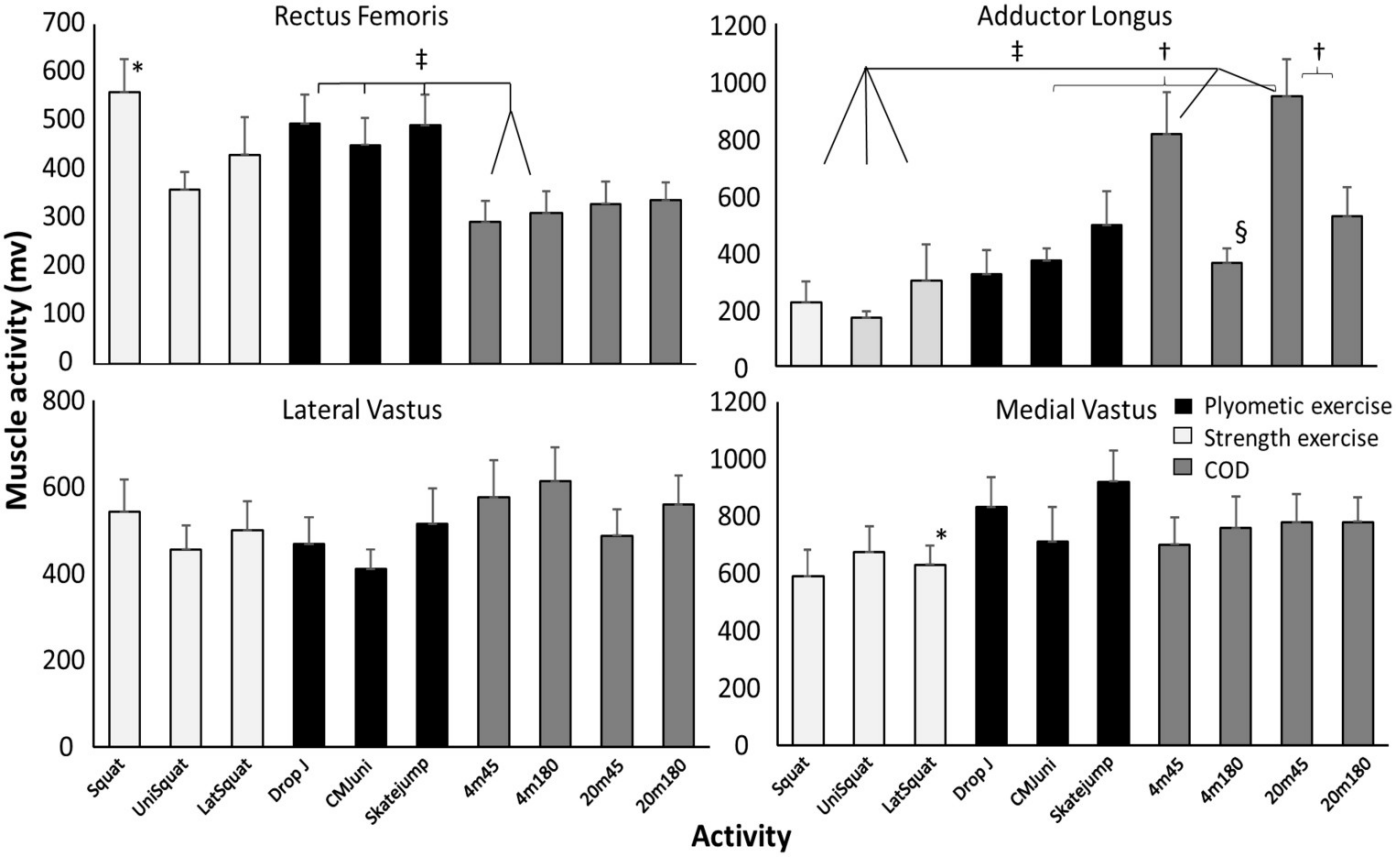
Peak (SEM) quadriceps and adductor longus activity during change of direction (COD) with a 45° or 180° turn from 4 and 20 m approach compared with different plyometric (unilateral CMJ, drop jump and skate jump) and strength (squat, lateral squat and unilateral squat) exercises averaged over all subjects. * indicates a significant difference (p<0.05) with all CODs. † indicates a significant difference (p<0.05) between these two variables. ‡ indicates a significant difference (p<0.05) between these CODs with these plyometric/strength exercises. § indicates a significant difference (p<0.05) with all other CODs.

Since no significant difference in EMG activity between the COD and acceleration step were found for any of the muscles, the peak muscle activity of these steps was used and compared with the different exercises. Only a significant difference in activity in the soleus and adductor longus muscles between the different CODs was found. Post hoc comparison revealed that the 4 m COD with 180° turn had lower activity than all other CODs and that the 20 m COD with a 45° turn had higher adductor longus activity than the 20 m COD with 180° turn. Soleus muscle activity with the 4 m COD with 180° turn was lower than those with a 20 m approach (Fig 4).

**Fig 5:**
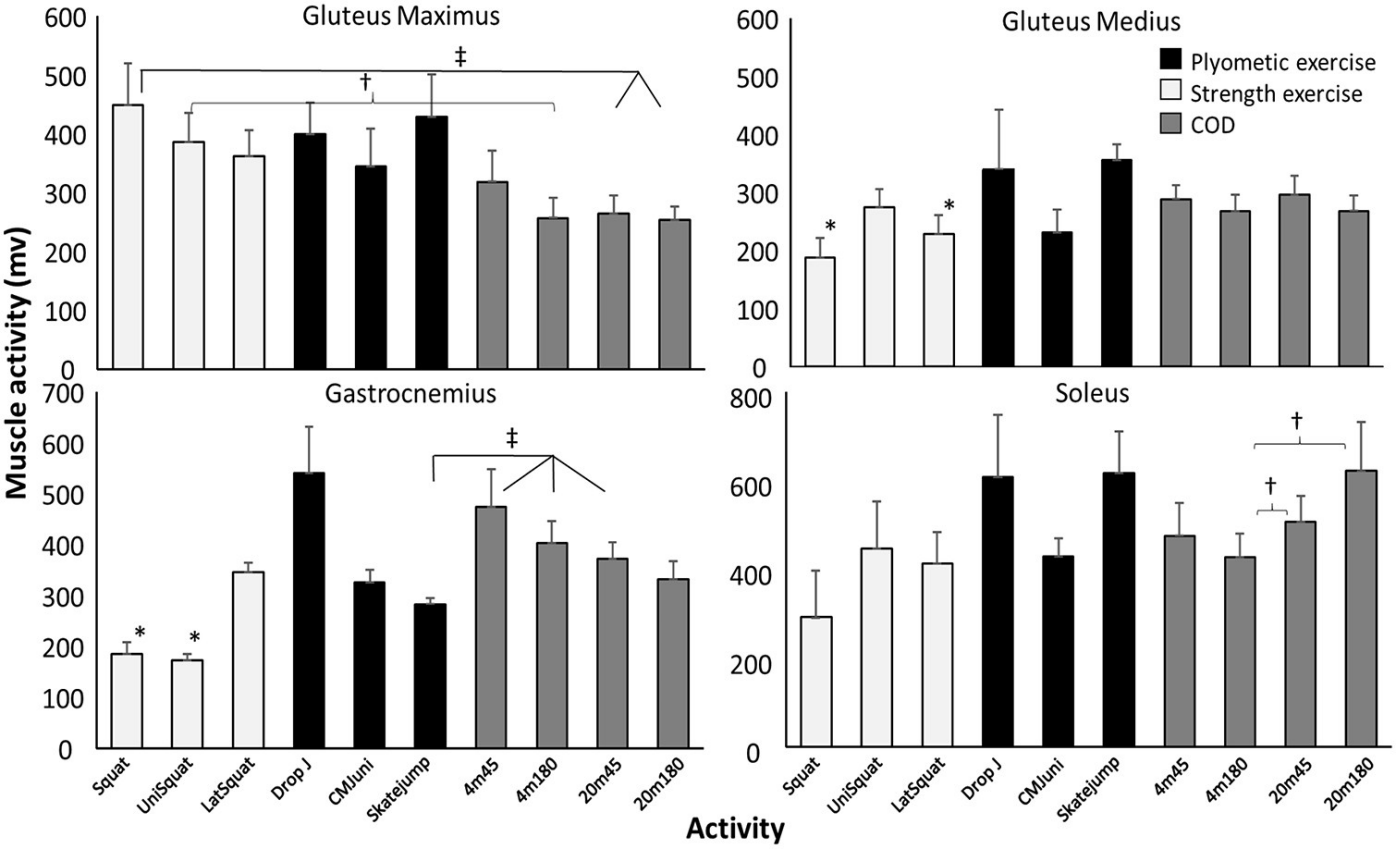
Peak (SEM) triceps surea and gluteus activity during change of direction (COD) with a 45° or 180° turn from 4 and 20 m approach compared with different plyometric (unilateral CMJ, drop jump and skate jump) and strength (squat, lateral squat and unilateral squat) exercises averaged over all subjects. * indicates a significant difference (p<0.05) with all CODs. † indicates a significant difference (p<0.05) between these two variables. ‡ indicates a significant difference (p<0.05) between these CODs with these plyometric/strength exercises.

**Fig 6:**
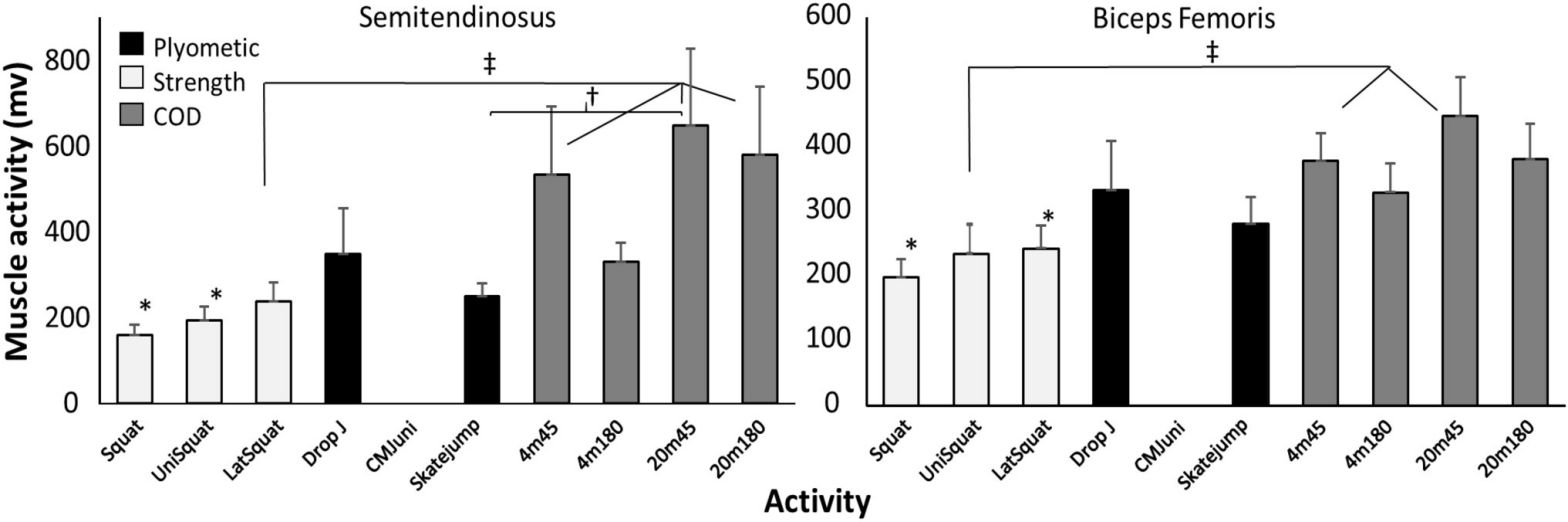
Peak (SEM) hamstring activity during change of direction (COD) with a 45° or 180° turn from 4 and 20 m approach compared with different plyometric (unilateral CMJ, drop jump and skate jump) and strength (squat, lateral squat and unilateral squat) exercises averaged over all subjects. * indicates a significant difference (p<0.05) with all CODs. † indicates a significant difference (p<0.05) between these two variables. ‡ indicates a significant difference (p<0.05) between these CODs with these plyometric/strength exercises.

In the bilateral squat exercise, less activity was found in most muscles compared with the different CODs, except for the rectus femoris and gluteus maximus, which had a higher activation in the squat exercise (Figs 4 and 5). Furthermore, rectus femoris activity in the plyometric exercises was higher than in CODs with a 4 m approach (Fig 4). The unilateral squat also showed significant lower activity in hamstring (Fig 6), adductor longus (Fig 4) and gastrocnemius (Fig 5) compared with most CODs, while gluteus maximus activity in this strength exercise showed higher activity with the 180° COD with a 4 m approach (Fig 5). Also, in the lateral squat exercise, lower gluteus medius, both hamstring muscles and medial vastus activation were found compared with CODs (Figs 4–6). From the plyometric exercises in the skate jump, lower gastrocnemius and semitendinosus activity were found compared with some CODs (Figs 5 and 6). In addition, in the unilateral countermovement jump (CMJ) for the adductor longus lower activity was shown compared with the 45° turn with 20m approach (Fig 4).

## Discussion

The main objective of the study was to investigate the association of performance measures in strength and plyometric tests with force- and velocity-dominant CODs. A secondary objective was to compare muscle activity of the different exercises with the CODs. None of the strength exercises correlated with COD performance. Peak muscle activation of the strength exercises was significantly different compared with the CODs, for several muscles, while only different muscle activity in the rectus femoris and gastrocnemius of the plyometric exercises was observed. Furthermore, the plyometric exercise performances revealed significant correlations with the CODs (Table 2).

### Correlations

The high correlations between plyometric performance and COD were expected and in accordance with previous research [42–45]. However, the low correlation between strength with COD performance was somewhat surprising, as strength qualities are factors influencing COD performance [15, 43], although strength is also context-specific. As the direction of forces produced in exercises should mimic the CODs [34], movements in the plyometric and strength tests were matched on bilateral vertical, unilateral vertical and lateral unilateral exercise to induce similar direction of forces produced and the muscles range of motion.

As specified per exercise, the skate jump correlated best with all CODs, which is explainable as it is the only exercise performed laterally and horizontal power productions are important for accelerating and decelerating in CODs [33, 39]. As COD movements occur unilaterally in the vertical/horizontal direction they require mostly medio-lateral force production [10]. Therefore, the unilateral countermovement jump also correlated with most CODs, but less due to the more vertical application of the jump. That is possibly also the reason why it was not related to the 20 m 180° COD, because this COD requires a greater amount of breaking and propulsive forces [36, 46] produced in both the anterior-posterior and medio-lateral direction [10]. The drop jump performance only correlated significantly with the 20 m 45° COD performance, which might be due to this COD performance being largely influenced by the straight-line sprint prior to the COD step [46–48]. This is because the reactive strength index is dictated by contact time, which also is also associated with sprint performance [49]. Time to exert a high amount of force in such fast dynamic movements is limited. Therefore, the relative amount of forces produced in the sprint prior to the COD step in the 20 m 45° COD is more vertically directed due to an increased velocity [50–52].

None of the strength tests induced a significant correlation with COD performance and this is perhaps related to the specificity of contraction velocity, which is suggested to be specific for both strength and sprinting [53, 54]. Probably the contractions are too slow to have a direct transfer to the different CODs. Furthermore, the pre-stretch of the muscles that may enhance the concentric contraction due to neural potentiation allows the recruitment of a greater number of motor units [55], with larger effect at increasing velocities [56, 57]. The faster pre-stretch in COD and plyometric exercises, thus attaining a higher level of cross-bridges may explain the higher observed muscle activation. However, an explanation of the muscle-specific tasks in the different conditions must not be neglected.

### Muscle activation

Firstly, both hamstring muscles revealed lower muscle activation in the strength exercises compared to the CODs (Fig 4). Knee flexor strength is suggested to help maintain trunk stability when decelerating in a COD [42] and producing horizontal force [58], which are important aspects for promoting overall COD performance [10, 59, 60]. As such, despite the relatively large emphasis on gluteus maximus and knee flexors in the different squat variations, the hamstrings are seemingly neglected. In comparison, the drop jump might induce a similar eccentric requirement of the hamstring muscles compared to the COD, as the hamstrings decelerate knee joint moments in the COD [61–63] prior to the concentric propulsive force [10]. The skate jump also revealed similar muscle activation of the hamstrings, which might be due to a faster movement increasing the eccentric load, utilizing a faster stretch-shortening cycle, in comparison to maximum lifts where the velocity is low.

Secondly, the strength exercises were found to induce lower muscle activation of the adductor longus and gluteus medius. Muscle activation was only similar for the gluteus medius when performing the unilateral squat, compared to the CODs. This might be due to the unilateral squat being performed on one foot, without a foot stabilizing, requiring more stabilization of the hip. The differences in the adductor longus muscles were only found for the force-dominant CODs. In the force-dominant CODs, the participants perform a total change in momentum [64] and start turning prior to the COD step [65] where the adductor functions as stabilizers of the hip [66, 67].

Thirdly, muscle activation in the gastrocnemius was found to be lower in the strength exercises when compared to the CODs, except for the lateral squat. This is because the lateral squat is the only strength exercise requiring plantar flexion of the ankle. On the other hand, all of the plyometric exercises require plantar flexion of the ankle, which explains the similar muscle activation to that in the CODs, as the ankle plantar flexes for the decelerating moment in the COD step [64, 67] by absorbing impact forces [59].

Based on the observed positive associations between the plyometric exercises and especially the skate jump with the CODs and observed peak muscle activation it seems that the plyometric exercises should be recommended to develop both force and velocity dominant CODs, while the measured strength exercises should not be recommended based upon the present findings. However, the present study did not investigate if implementing these exercises over a period of time helps the COD performance. Earlier training studies have shown that implementing some of these plyometric exercises in training increased COD performance [34]. The skate jump had the highest associations with the different COD, while only similar exercises performed laterally are supplemented in earlier training interventions [16, 68, 69], it would be interesting to investigate the isolated effect of this exercise in future training studies and how it effects COD performance. Furthermore, the plyometric exercises displayed a greater similarity in muscle activation in the hamstring muscles, adductor longus, gastrocnemius, gluteus medius and a greater association with COD performance compared to the strength exercises. It is possible that strength exercises did not show the same level of association due to the lack of activation in these muscles. As such, it is possible that selecting different strength exercises or modifying the current strength exercises with the goal of stimulating muscle activation more like the COD can better improve COD performance.

### Limitations

Although the plyometric exercises are seemingly more specific towards both force- and velocity-dominant CODs, some limitations must be addressed. Firstly, the strength exercises were measured at maximal external load lifted, possibly inhibiting the specificity towards the CODs. A lighter load with the intention of maximizing power output might reveal more correlation in performance measures [19]. Secondly, the number of tested players and wide variety of player level (second to sixth national level) could be a limitation to generalise the findings. But the variation in test performances between the players was low and thereby these performances are comparable for players of different levels. However, more studies with players from same playing level should be performed to investigate if the associations between CODs with strength and plyometric tests are comparable with the ones found in the present study. Thirdly, EMG measurements are always at risk of crosstalk between muscles, especially the fast-dynamic movements like the plyometrics and CODs. Future research should be conducted with a force plate in the COD step and for the different strength and plyometric tests, to investigate the forces produced. These force measurements could provide greater insight into the specificity of the strength and plyometric exercises towards CODs, especially the exercises performed laterally.

## Conclusion

The plyometric tests revealed several associations with performance measures in both the force- and velocity-dominant CODs, whereas the strength tests failed to provide such an association, due to the slow contraction velocity of maximal lifts. Due to the observed differences in muscle activation, plyometric exercises are indicated to share more physical similarities with both the strength- and velocity-dominant CODs, in comparison to the strength exercises in this cohort. Thus, as a practical application it is suggested, when supplementing COD-specific training with extra training, to use the studied plyometric exercises rather than the strength exercises based upon similar muscle use and associations with velocity- and force-dominant CODs.

## Supporting information

S1 Data (PDF)
