# Supplementary material for: Association of strength and plyometric exercises with change of direction performances
Source: PLoS One. 2020 Sep 10;15(9):e0238580. doi: 10.1371/journal.pone.0238580 (PMC7482936; doi:10.1371/journal.pone.0238580)
Supplement: S1 Data — (PDF) [file pone.0238580.s001.pdf]

Subjects	Biometrics			Wilks score			Str
	Age	BodyMasskg	BodyHeight	Squat	UniHalfSquat	LatSquat	Squat
1	23	61	165	92.42	95.78	92.42	110
2	25	83	182	73.42			110
3	22	85	187	71.75		65.83	109
4	18	72	182	95.38	72.64	73.37	130
5	19	66	186	70.67	65.96	62.82	90
6	23	71	170	81.55	69.69	59.31	110
7	23	81	177	94.84	70.45	74.51	140
8	25	80	179	105.82	71	81.92	155
9	24	75.5	183	81.57	73.77	85.12	115
10	25	80	179	88.75	81.24	81.92	130
11	18	85.8	189	88.41	64.83	65.49	135
12	25	85	188	75.7	71.75	59.25	115
13	23	81	177	101.61	80.61	81.29	150
14	25	80	179	100.7	72.71	78.51	147.5
15	24	74.2	183	82.56	67.48	64.61	115
16	25	82	179	80.69	53.12	60.52	120
17	18	87	188	87.74	70.84	64.99	135
18	25	84	188		72.25		
19	23	102.2	180				
20	19	75	185				
21	19	82.1	185	87.35	69.88	77.27	130
22	23	72	171	102.72	90.98	66.03	140
23	24	93.5	189	81.46	66.73	62.66	130
**average**	**22.52**	**79.93**	**181.35**	**87.26**	**72.72**	**71.47**	**125.83**
**SD**	**2.61**	**8.76**	**6.40**	**10.58**	**9.39**	**9.98**	**16.62**

ength (lifted in kg)		Strength (kg/bodymass			Plyometric exerci		
UniHalfSquat	LatSquat	Squat	UniHalfSquat	LatSquat	DJct	Djheight	DJRSindex
114	110	1.80	1.87	1.80	216	41.3	1.91
		1.33			200	27.6	1.38
	100	1.28		1.18	212	293	1.38
99	100	1.81	1.38	1.39	212	39	1.84
84	80	1.36	1.27	1.21	219	24.9	1.14
94	80	1.55	1.32	1.13			
104	110	1.73	1.28	1.36	187	18	0.96
104	120	1.94	1.30	1.50	203	26.7	1.32
104	120	1.52	1.38	1.59	208	30.8	1.48
119	120	1.63	1.49	1.50	191	26.2	1.37
99	100	1.57	1.15	1.17	249	33.4	1.34
109	90	1.35	1.28	1.06	255	29.4	1.15
119	120	1.85	1.47	1.48	272	29	1.06
106.5	115	1.84	1.33	1.44	220	37.9	1.72
94	90	1.55	1.27	1.21	208	30.5	1.47
79	90	1.46	0.96	1.10	217	24.3	1.12
109	100	1.55	1.25	1.15	198	32.6	1.65
109			1.30		172	31.1	1.81
					228	26.4	1.16
					193	28.7	1.49
104	115	1.58	1.27	1.40			
124	90	1.94	1.72	1.25	178	30.9	1.74
106.5	100	1.39	1.14	1.07	225	31.6	1.41
**104.26**	**102.63**	**1.60**	**1.34**	**1.31**	**212.52**	**42.54**	**1.42**
**11.33**	**13.58**	**0.21**	**0.20**	**0.20**	**24.52**	**57.62**	**0.27**

ise Performances							
SquatJumpUni	Skatmax	SkatJmpCt	tid4m45	tid4m180	tid20m45	tid20m180	part45
20	180		167	245	400	487	144
18.2	201	430	134	241	373	510	132
14.6			160	241	373	417	123
27.6	231	453	153	218	366	484	119
17.6	188	461	186	242	420	512	127
			197	257	432	528	140
13.8	184		182	263	420	516	137
15.6	218	474	162	237	396	478	124
19.1	201	496	158	241	397	496	126
16.1	191		163	244	411	483	147
18.1	210	602	179	259	414	491	153
19.6	212	349	147	220	394	492	131
20.2	200	585	160	239	407	482	142
19.1	231	493	161	235	371	447	125
19.4	201	546	160	246	420	503	149
15.3	191	592	183	265	417	502	148
22.3	220	693	177	251	393	473	145
21.6	227	342	147	224	373	479	123
16.2	163	441	177	251	442	518	154
15.7	188	370	178	243	399	483	133
	194	387	168	231	392	463	133
14.5	170	304	184	257	416	498	142
22.3	220	511	174	244	396	487	128
**18.42**	**201.00**	**473.83**	**167.70**	**243.22**	**400.96**	**488.22**	**135.87**
**3.32**	**19.25**	**103.46**	**14.96**	**12.54**	**20.51**	**24.13**	**10.61**

part180
220
257
213
238
219
242
233
212
231
217
223
233
222
206
231
222
223
225
242
213
210
227
216
**225.00**
**12.15**
